# Does type 2 diabetes duration influence the effectiveness of an aerobic exercise intervention: Results from the INTENSITY study

**DOI:** 10.1371/journal.pone.0304341

**Published:** 2024-06-06

**Authors:** Amy M. Thomson, Brittany V. Rioux, Travis J. Hrubeniuk, Danielle R. Bouchard, Martin Sénéchal

**Affiliations:** 1 Cardiometabolic Exercise & Lifestyle Laboratory, Fredericton, Canada; 2 Faculty of Kinesiology, University of New Brunswick, Fredericton, Canada; 3 CancerCare Manitoba, Canada; 4 Community Health Sciences, Max Rady College of Medicine, University of Manitoba, Winnipeg, Canada; Hamasaki Clinic, JAPAN

## Abstract

**Background:**

Studies suggest that longer durations of T2DM increase the risk of T2DM complications and premature mortality. However, whether T2DM duration impacts the efficacy of an aerobic exercise intervention is unclear.

**Objective:**

The purpose of this study was: 1) to compare changes in body composition, cardiorespiratory fitness, and glycemia between individuals with short- and long-duration T2DM after aerobic exercise and 2) to determine whether these changes were associated with changes in glycemia by T2DM duration.

**Methods:**

A secondary analysis of the INTENSITY study (NCT03787836), including thirty-four adults (≥19 years) with T2DM who participated in 28 weeks of aerobic exercise training for 150 minutes per week at a moderate-to-vigorous intensity (4.5 to 6.0 metabolic equivalents (METs)). Using pre-established cut-points, participants were categorized into two groups 1) short-duration T2DM (<5 years) or 2) long-duration T2DM (≥5 years). Glycemia was measured by glycated hemoglobin (HbA1c), body composition by BodPod, and cardiorespiratory fitness by a measure of peak oxygen consumption (VO_2peak_). All measurements were performed at baseline, 16 weeks, and 28 weeks.

**Results:**

Participants in the short-duration T2DM group experienced decreases in fat mass (kg) (*p* = 0.03), HbA1c (*p* = 0.05), and an increased relative VO_2peak_ (*p* = 0.01). Those with long-duration T2DM experienced decreases in fat mass (kg) (*p* = 0.02) and HbA1c (*p* <0.001) and increased fat-free mass (*p* = 0.02). No significant differences were observed between groups in any outcomes. Changes in fat mass (*r* = 0.54, *p* = 0.02), and body fat percentage (*r* = 0.50, *p* = 0.02) were significantly associated with the change in HbA1c in those with a long-duration T2DM only.

**Conclusion:**

Our results suggest T2DM duration did not differently impact the efficacy of a 28-week aerobic exercise intervention. However, changes in body composition were associated with better glycemia in individuals with longer T2DM duration only.

## Introduction

Type 2 diabetes mellitus (T2DM) is a growing epidemic characterized by chronic hyperglycemia affecting more than 480 million individuals globally [1], and is expected to surpass 700 million individuals by 2045 [1]. T2DM is associated with several microvascular and macrovascular complications [2–7], with approximately 50% of patients experiencing complications at the time of diagnosis [8, 9].

An underappreciated risk factor for managing T2DM and its complications is diabetes duration. Longstanding T2DM increases the risks of premature mortality, complications, and multimorbidity [7, 10–14]. Moreover, the risk of premature mortality has been shown to increase by 23% in individuals who have lived with T2DM for more than 5 years and 36% in individuals who have lived with T2DM for more than 10 years [13]. The risk of microvascular and macrovascular complications also increases with diabetes duration by 28% and 13%, respectively, for each 5-year increase in T2DM duration [7]. Multimorbidity is similarly affected as a 10-year T2DM duration is associated with a five-fold increased risk of living with 5 or more comorbidities. [14]. The impacts of increased T2DM duration extend to exercise capacity as increasing T2DM duration has been associated with impaired cardiorespiratory fitness, decreased exercise tolerance, and progressive declines in muscle strength [15, 16].

Although the initial treatment approach for T2DM and its complications is lifestyle modification, longstanding T2DM has been associated with decreased effectiveness of various lifestyle interventions [17–21]. Recent Diabetes Canada guidelines also highlight the potential role of disease duration in lifestyle modifications with a decreased likelihood of achieving T2DM remission with longer disease duration [22]. As such, T2DM duration could be a relevant factor to consider when prescribing exercise interventions for T2DM management. For instance, lipotoxicity can contribute to beta cell dysfunction [23] and longer T2DM durations are associated with increased intrapancreatic fat, contributing to further beta cell damage [24]. As exercise interventions can reduce pancreatic fat [25] and decrease whole body fat, prescribing interventions to individuals earlier on in their lifetime after diagnosis may assist in slowing disease progression. However, it remains unclear whether T2DM duration influences exercise intervention efficacy outside of the scope of T2DM remission. A study by Tan et al. (2012) demonstrated significant improvements in body fat percentage and HbA1c among older adults with long-duration T2DM (> 10 years) following a six-month aerobic and resistance training intervention, showing the benefits of an exercise intervention for individuals with longstanding T2DM [26]. However, Tan et al. (2012) did not compare the observed changes with individuals of a shorter disease duration [26]. To the best of our knowledge, only one study has directly compared the impact of short- and long-duration T2DM on exercise training efficacy [27]. Following 12 weeks of resistance training, the participants of both T2DM duration groups significantly decreased body mass, body mass index (BMI), waist circumference, fat mass, body fat percentage, and HbA1c; however, no differences were observed between groups [27].

Most studies investigating the impact of T2DM duration thus far focus on surgical outcomes [28, 29], low-calorie diets [30, 31], or include disease duration solely as a baseline characteristic and confounding factor [32–34]. As such, studies that compare the impact of short- and long-duration T2DM on exercise intervention efficacy: 1) are scarce, 2) do not investigate aerobic exercise alone, 3) include only female participants, and 4) do not investigate if changes in body composition and cardiorespiratory fitness are associated with a change in glycemia among different T2DM duration groups.

Therefore, the purpose of this analysis was twofold: 1) to compare the changes in body composition, cardiorespiratory fitness, and HbA1c between individuals with short- and long-duration T2DM who participated in 28 weeks of aerobic exercise, and 2) to investigate if these changes in body composition and cardiorespiratory fitness were associated with improved glycemia. We hypothesized that the short-duration T2DM group would significantly reduce HbA1c and enhance body composition compared to the long-duration T2DM group. We also hypothesized that changes in body composition would be associated with changes in HbA1c.

## Materials and methods

This study is a secondary analysis of the Improving Individual Glycemic Response with Exercise Intensity (INTENSITY) study (NCT03787836). The INTENSITY study was a two-phase, 28-week double-blind randomized trial investigating the impact of increasing exercise intensity on responder status. Written informed consent was obtained from all participants in the INTENSITY study. More details on the methods have been published elsewhere [35].

### 2.1 Participants

Participants were recruited from April 1^st^, 2019, to November 30^th^, 2020, in Fredericton and surrounding areas in New Brunswick, Canada, using advertisements placed in local businesses and social media. Recruitment also took place through newsletters to students and staff at the University of New Brunswick, St. Thomas University, and medical diabetes clinics.

Inclusion criteria for participants in this secondary analysis were: 1) aged 19 years or older, 2) currently living with self-reported T2DM or T2DM confirmed with HbA1c measurements, and 3) not currently partaking in any regular physical activity (i.e., consistent participation in organized programs, or averaging 10,000 steps per day (PiedzoRX) over a seven-day period at baseline).

**Fig 1:**
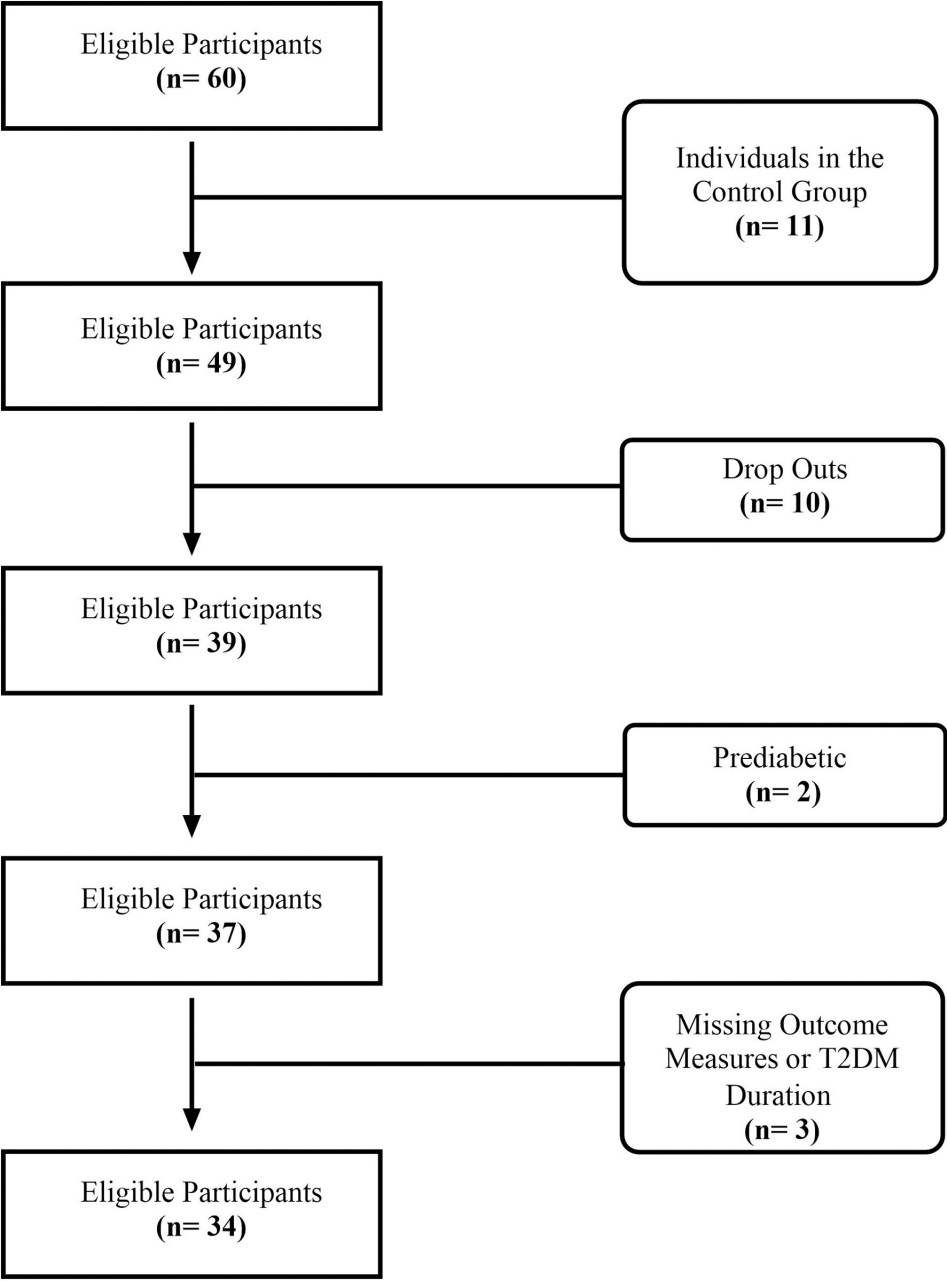
Participant flow chart.

Exclusion criteria for participants in this secondary analysis were: 1) diagnosed with low iron concentrations or anemia, 2) diagnosed with any red blood cell altering conditions, 3) currently living with a cardiovascular disease that would impact the safety of participation in exercise training, 4) currently prescribed medications that could impact the use of a heart rate monitor for accurate intensity tracking, 5) prediabetic, or 6) had missing primary and secondary outcome measure data. A total of 60 participants were recruited for the INTENSITY study; participants excluded from this secondary analysis were in the control group (n = 11), dropped out of the intervention (n = 10), had pre-diabetes (n = 2), or had missing data for T2DM duration or outcome measures (n = 3) (Fig 1).

### 2.2 Aerobic exercise training

#### Phase 1

The INTENSITY trial occurred in two phases [35]. Participants allocated to the intervention group engaged in 16 weeks of supervised exercise at 4.5 metabolic equivalents (METs) on a treadmill. The exercise protocol began with a 4-week progressive start involving 80 minutes of exercise at 4.5 METs in week 1, 100 minutes in week 2, 120 minutes in week 3, and 135 minutes of exercise in week 4, followed by 150 minutes of exercise per week at 4.5 METs for the remaining 12 weeks of the intervention period. Participants choose the frequency (number of weekly sessions) needed to complete the exercise time (150 minutes/week) as long as the total number of sessions per week was greater than one. Participants were also allowed to choose the grade and speed of the treadmill during the exercise time, as long as the prescribed absolute intensity was achieved and maintained throughout the sessions. Each training session started with a 5-minute warmup on the treadmill to achieve the required intensity (4.5 METs), which was not counted in the total exercise time. Participants were re-evaluated every four weeks to account for cardiorespiratory improvements and ensure appropriate training intensity.

#### Phase 2

Participants in the intervention group were randomly allocated to either a maintained or increased intensity group. Participants allocated to the maintained intensity group continued the 150 minutes of aerobic exercise at an intensity of 4.5 METs for 12 weeks. The participants allocated to the increased intensity group increased their 150 minutes of aerobic exercise from an intensity of 4.5 METs to 6.0 METs for 12 weeks. Participants scheduling, speed, slope, and supervision followed the same methods as Phase 1.

### 2.3 Anthropometric and body composition measures

Physiological and anthropometric measurements occurred at baseline, 16 weeks, and 28 weeks. Participant height, blood pressure, and waist circumferences were measured by a research team member following the Canadian Society for Exercise Physiology (CSEP) protocols [36]. The BodPod was used to estimate body composition measures (body mass, fat mass, and fat-free mass) following a 12-hour overnight fast, using air displacement plethysmography to calculate body density. Body mass index (BMI) was calculated using the following formula BMI = weight (kg)/ height (m^2^).

### 2.4 Cardiorespiratory fitness measures

Cardiorespiratory fitness was assessed by peak oxygen consumption (VO2peak) using a modified Balke and Ware treadmill test protocol. In this modified protocol, participants walked at 3.4 miles per hour (mph) at a 0% incline on a treadmill, and after 2 minutes, the grade was increased to 5.0%. The grade was then progressively increased by 1.0% every minute until 15.0% grade was achieved. If the participant was not fatigued, the grade was maintained, and the speed was then increased by 0.5mph each minute until volitional fatigue. A TrueOne 2400 Metabolic Cart and Polar FT1 heart rate monitor were used to continuously record gas exchange and heart rate, respectively. Treadmill time to exhaustion (TTE) was determined from the VO2peak test performed at baseline and follow-up. TTE was reported as the maximal time for the overall test duration.

### 2.5 Glycemia measures (HbA1c)

Glycemia was measured at baseline, 16 weeks, and 28 weeks using a DAC Vantage Analyzer. A finger prick was performed using a Safe-T Pro Plus single-use lancet to collect a 1 microliter sample of whole blood. A rapid assessment of HbA1c was conducted by loading the sample into the DCA Vantage Analyzer, and the results were recorded after approximately 6 minutes. To increase reliability, HbA1c was measured twice at each time point, less than seven days apart, with the mean value used for analysis.

Information about T2DM medications was self-reported by participants and recorded on a medication history sheet. Medication changes were recorded as follows: 0 = reduced or discontinued, 1 = no change, and 2 = increased. Reduced/discontinued medications and no change were collapsed together since they represent a positive effect compared to increased medication.

### 2.6 Diabetes duration measure

Participant T2DM duration was self-reported during baseline testing and recorded as time in months since clinical diagnosis. Since there was no significant effect of the intervention in the original INTENSITY study, the participants in the maintained and intensity groups were combined for analysis purposes (p > 0.05). Thereafter, participants were categorized as follows: short-duration T2DM: T2DM duration < 5 years and long-duration T2DM: T2DM duration ≥5 years. Although there are no universally accepted cut-points for diabetes duration, a cut-point of 5 years was selected based on 1) previous study using this number of years [11], 2) studies showing a strong increased risk of premature mortality [7, 11, 13], and finally, because this cut-points will allow us to compare our results with pre-existing literature.

### 2.7 Statistical analysis

The normality of the data was assessed using the Kolmogorov–Smirnov test, and a visual inspection of the data was performed. Continuous and categorical variables were presented as mean ± standard deviation (SD) or n (%). Repeated measures analyses of variance (ANOVAs) within-between groups were used to investigate the impact diabetes duration over time on primary outcome measures and reported as F-values and p-values. The analysis was then adjusted for baseline differences in fat mass between the two groups. The HbA1c analysis was also adjusted for changes in T2DM medications. Pearson’s correlations were performed to determine if the changes in body composition were associated with a change in HbA1c. The significance level was accepted at p < 0.05, and all analyses were performed using IBM SPSS statistics version 22.0.

## Results

### 3.1 General characteristics

At baseline, the short- and long-duration T2DM cohorts differed in average age, T2DM duration, fat mass, and HbA1c. The short-duration cohort had an average age of 52.6 ± 6.3 years and T2DM duration of 2.4 ± 1.2 years, whereas the long-duration cohort had an average age of 61.2 ± 9.40 years and T2DM duration of 13.04 ± 5.6 years. For fat mass and HbA1c, the short- and long-duration groups averaged 44.8 ± 11.0kg and 6.4 ± 0.53%, and 33.9 ± 11.0kg and 7.3 ± 0.79% respectively. All general characteristics of the participants for baseline, 16 weeks, and 28 weeks are described in Table 1.

**Table 1:** Baseline characteristics of the participants.

	Short T2DM Duration <5 years n = 12	Long T2DM Duration ≥5 years n = 22
Age (years)	52.6 [48.6, 56.6]	61.2 [57.0, 65.4]*
Diabetes duration (years)	2.4 [1.7, 3.2]	13.0 [10.6, 15.5] *
Medications (#)	1.1 [0.5, 1.7]	2.0 [1.5, 2.4]
Sex (males %)	8 (53.3)	13 (59.1)
Weight (kg)	98.9 [91.4, 106.4]	91.6 [82.1, 101.1]
BMI (kg/m^2^)	34.7 [31.4, 38.0]	30.8 [28.3, 33.4]
Waist circumference (cm)	113.4 [107.5, 199.3]	110.3 [103.9, 116.7]
Body fat (%)	43.9 [38.5, 49.4]	37.2 [33.5, 40.9]*
Total fat mass (kg)	44.8 [37.8, 51.8]	33.9 [28.9, 39.0]*
Fat free mass (kg)	54.9 [50.6, 59.2]	56.8 [50.3, 63.4]
HbA1c (%)	6.4 [6.1, 6.7]	7.4 [7.1, 7.8]*
TTE (s)	766.3 [601.4, 936.8]	829.7 [716.9, 908.7]
Relative VO_2 peak_ (mL·kg^-1^·min^-1^)	24.0 [20.3, 27.9]	25.4 [22.8, 27.9]

Data reported as mean and 95% confidence intervals for continuous variables and n (%) for categorical variables. The * represents significant differences between groups.

### 3.2 Changes in anthropometric and body composition characteristics

The short-duration T2DM group displayed a significant reduction in weight (kg) (*F*(2) = 4.342, *p* = 0.03), BMI (*F*(2) = 4.659, *p* = 0.02), waist circumference (cm) (*F*(2) = 6.150, *p* = 0.008), and fat mass (kg) (*F*(2) = 4.003, *p* = 0.03), following the completion of the 28-week trial. The long-duration T2DM group experienced a significant decrease in fat mass (*F*(2) = 5.571, *p* = 0.01) and body fat percentage (*F*(2) = 9.243, *p =* 0.002), and an increase in fat-free mass (*F*(2) = 3.641, *p* = 0.046). These changes in body composition characteristics can be observed in Table 2. No interaction effect (time x T2DM duration) was found when the analysis was conducted either unadjusted or adjusted for differences in baseline percent body fat or fat mass.

**Table 2:** Intervention effects on short and long T2DM duration groups.

**Short T2DM Duration** **<5 years**	**Baseline**	**16-Weeks**	**28-Weeks**	**p-value**
Age (years)	52.6 [48.6, 56.6]	--	--	--
Sex (males %)	6 (50)	--	--	--
Weight (kg)	98.9 [91.4, 106.4]	97.7 [90.5, 104.9]	97.3 [90.4, 104.2]	**0.03**
BMI (kg/m^2^)	34.7 [31.4, 38.0]	34.3 [32.0, 37.6]	34.1 [30.9, 37.3]	**0.02**
Waist circumference (cm)	113.4 [107.5, 199.3]	111.4 [105.4, 117.4]	110.7 [104.5, 116.8]	**<0.01**
Body fat (%)	43.9 [38.5, 49.4]	42.9 [37.4, 48.3]	42.7 [37.2, 48.2]	0.13
Total fat mass (kg)	44.8 [37.8, 51.8]	42.4 [35.0, 49.9]	42.0 [34.7, 49.3]	**0.03**
Fat free mass (kg)	54.9 [50.6, 59.2]	55.2 [51.1, 59.4]	55.3 [50.1, 59.6]	0.80
HbA1c (%) †	6.4 [6.1, 6.7]	6.2 [5.7, 6.7]	6.4 [6.0, 6.8]	**0.05**
TTE (s)	766.3 [601.4, 936.8]	990.3 [925.7, 1079.2]	997.2 [911.5, 1082.8]	**<0.01**
Relative VO_2 peak_ (mL·kg^-1^·min^-1^)	24.0 [20.3, 27.9]	24.4 [21.9, 27.6]	26.7 [23.0, 30.5]	**0.01**
**Long T2DM Duration** **≥5 years**	**Baseline**	**16-Weeks**	**28-Weeks**	**p-value**
Age (years)	61.2 [57.0, 65.4]	--	--	--
Sex (males %)	13 (59.1)	--	--	--
Weight (kg)	91.6 [82.1, 101.1]	90.9 [81.6, 100.2]	89.9 [81.3, 98.5]	0.10
BMI (kg/m^2^)	30.8 [28.3, 33.4]	30.6 [28.0, 33.1]	30.3 [28.0, 32.6]	0.10
Waist circumference (cm)	110.3 [103.9, 116.7]	109.4 [103.2, 115.6]	109.3 [103.9, 114.7]	0.32
Body fat (%)	37.2 [33.5, 40.9]	36.1 [32.3, 39.4]	35.9 [32.0, 39.1]	**<0.01**
Total fat mass (kg)	33.9 [28.9, 39.0]	33.1 [27.6, 37.5]	32.4 [27.4, 36.3]	**0.02**
Fat free mass (kg)	56.8 [50.3, 63.4]	57.6 [51.1, 64.0]	57.4 [51.1, 63.7]	**0.02**
HbA1c (%) †	7.4 [7.1, 7.8]	7.2 [6.9, 7.5]	7.0 [6.7, 7.3]	**<0.01**
TTE (s)	829.7 [716.9, 908.7]	972.8 [904.4, 1019.7]	1018.4 [966.9, 1069.8]	**<0.01**
Relative VO_2 peak_ (mL·kg^-1^·min^-1^)	25.4 [22.8, 27.9]	27.0 [24.0, 29.2]	27.2 [24.8, 29.5]	0.06

Data reported as mean with 95% confidence intervals for continuous variables and n (%) for categorical variables

† HbA1c adjusted for medication changes.

### 3.3 Changes in cardiorespiratory fitness measures

The short-duration T2DM cohort demonstrated an increase in relative VO2peak (*F* (2) = 5.302, *p* = 0.01) and TTE (*F* (2) = 16.439, *p* < 0.001) throughout the intervention. The long-duration T2DM cohort increased TTE (*F* (2) = 26.843, *p* < 0.001). These changes in cardiorespiratory fitness measures can be observed in Table 2.

### 3.4 Changes in glycemia

A significant time effect was observed in both the short (*F* (2) = 3.448, *p* = 0.05) and long (*F* (2) = 12.782, *p* = <0.001) duration T2DM groups when adjusted for changes in T2DM medications over the intervention. The repeated measures ANOVA adjusted for T2DM medication revealed a time effect of the intervention on glycemia (*F* (2) = 5.768, *p* = 0.005) when adjusting for the change in T2DM medication, but no interaction effect (time x group) was observed.

### 3.5 Associations of glycemia and body composition

**Fig 2:**
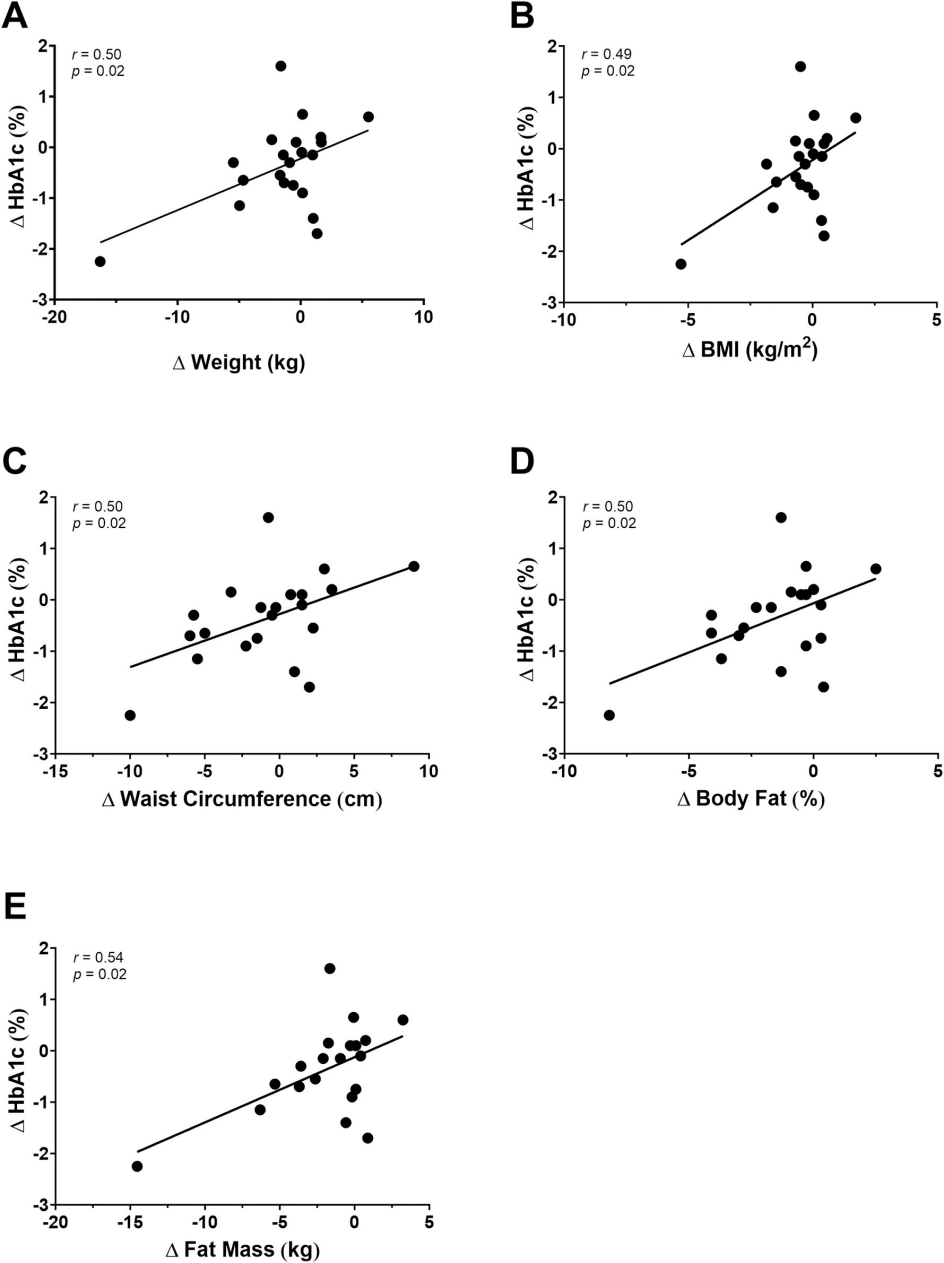
A-E: Correlations between changes in anthropometric measures, body composition and changes in glycemia in individuals with long-duration T2DM.

No significant correlations were found between changes in HbA1c and changes in body composition after the 28-week intervention for the short-duration group. The long-duration T2DM group experienced significant associations with the changes in HbA1c and weight (*r* = 0.50; *p* = 0.02), BMI (*r* = 0.49; *p* = 0.02), waist circumference (*r* = 0.50, *p* = 0.02), fat mass (*r* = 0.54; *p* = 0.02) and body fat percentage (*r* = 0.50; *p* = 0.02; Fig 2A–2E).

## Discussion

This secondary analysis explored whether changes in glycemia, cardiorespiratory fitness, and body composition differed among individuals with short- or long-duration T2DM following an aerobic exercise intervention, and whether these changes were associated with improved glycemia. Despite the lack of group differences in body composition measures and cardiorespiratory fitness, we found that changes in several of these measures were significantly improved within the short- and long-duration T2DM groups. We also found that changes in anthropometric measures and fat mass were associated with greater changes in glycemia for individuals with long-duration T2DM. No significant differences were observed between the two T2DM duration groups for changes in body composition. However, a moderate, positive correlation was observed between changes in fat mass and glycemia in the long-duration T2DM group, suggesting that individuals who have lived with T2DM for at least 5 years may improve glycemia with smaller changes in fat mass. Given that people are receiving T2DM diagnoses earlier in life, and therefore live with the disease for longer, these findings may help guide T2DM management for those with longstanding diabetes.

Our study shows that a 28-week moderate-to-vigorous intensity, supervised, aerobic exercise intervention changes body composition for those with long- and short-duration T2DM with no differences between groups. The short-duration T2DM group experienced significant changes in weight, body mass index, waist circumference, and fat mass. These results are consistent with other data on the benefits of aerobic exercise in individuals with T2DM [37–39]. Likewise, Park et al. (2016) [27] highlighted how resistance training may also improve anthropometric measures for women with short-duration T2DM. However, Park et al. (2016) [27] observed significant improvements in body fat percentage (-5.9%) in their short-duration T2DM group, which was not observed in the present study. The difference between the results observed in the Park et al. (2016) [27] study and our study could be explained by differences in exercise intensity, frequency, and/or modality [40, 41]. In the Park et al. (2016) [27] study, participants performed a higher frequency and intensity of exercise with two daily resistance training sessions five days per week, while our participants performed 150 minutes of aerobic exercise over 2–4 days per week. Nevertheless, our study adds to the available evidence on T2DM duration and demonstrate that a lower dose of aerobic exercise alone can elicit meaningful changes in body composition for those with short-duration T2DM.

The long-duration T2DM cohort experienced a significant decrease in fat mass and body fat percentage, consistent with the benefits typically anticipated following participation in aerobic exercise [37, 38, 42]. These changes are similar to the 1.3% reduction reported by Tan et al. (2012) [26], and are in line with the 4.5% decrease in body fat percentage in the long-duration T2DM participants observed by Park et al. (2016) [27]. However, in their study [27] the participants with long-duration T2DM observed significant changes in other anthropometric measures, including weight, body mass index, and waist circumference. Similar to the results in the short-duration T2DM cohort, the differences in exercise modality, frequency, and intensity between these studies could explain the varying degrees of improvements. It is of note that the average duration of T2DM for our long-duration participants (13 years) is comparable to the average of those in the Park et al. (2016) [27] study (10 years) and the Tan et al. (2012) [26] study (16.7 years). Although the exercise interventions differed between studies, the participants with long-duration T2DM reported improvements in body composition following all three interventions. Our results demonstrate that a solely aerobic intervention can be beneficial for those with long-duration T2DM to decrease body fat percentage.

The significant increase in TTE observed in both the short-and long-duration T2DM cohorts is supported by aerobic exercise’s capacity to increase cardiorespiratory fitness levels [43]. The significant effect of the intervention on cardiorespiratory fitness in the long-duration T2DM group is further corroborated by a similar increase in cardiorespiratory fitness observed in the long-duration cohort in the Park et al. (2016) [27] study. Interestingly, the short-duration T2DM participants in the Park et al. (2016) [27] study did not experience the same significant cardiorespiratory fitness improvements as their long-duration group. However, in the current study, a significant improvement was observed for both groups despite a lack of interaction between time and T2DM duration for changes in cardiorespiratory fitness. Our intervention demonstrates that 150 minutes of moderate-to-vigorous aerobic exercise per week can elicit meaningful changes in cardiorespiratory fitness, regardless of T2DM duration. These results add to the existing data by providing insight into the efficacy of a 28-week aerobic exercise intervention on cardiorespiratory benefits for individuals with long-and short-duration T2DM.

The changes in glycemia in the present study were significant in both the short-and long-duration T2DM cohorts with long-duration T2DM being the only group showing a dose-response manner reduction in HbA1c. However, correlations were only observed between the changes in HbA1c and several body composition measures for the long-duration T2DM cohort. These results align with findings from Tan et al. (2012) [26] who found a significant change in HbA1c (-0.55%) for individuals with long-duration T2DM and correlations with waist-to-hip ratio and the 2-hour post-glucose challenge serum insulin. Park et al. (2016) [27] found significant changes in HbA1c for both their long (-1.37%) and short (-0.93%) duration T2DM groups. The glycemia changes in these past studies were greater than those observed in the current study; this difference could be influenced by the exercise modality, as some studies have found that resistance training and combined aerobic and resistance training have an additive effect on glycemia [44–47]. Nevertheless, our results suggest that for individuals with longstanding T2DM, 150 minutes of aerobic exercise per week can result in changes in glycemia. These results highlight the importance of aerobic interventions for glycemia in the long-duration cohort and could have implications for T2DM management as individuals with longstanding T2DM duration (>5 years) have shown increased risks of premature mortality, especially in those with worse glycemia [11].

The current study has a few limitations that must be taken into consideration. First, this study has a small sample size and was not originally powered to answer this research question [48]. Second, T2DM duration was self-reported by participants, which could have impacted our results as self-reported data is known to be affected by recall bias. Third, many of the participants in this study had well-controlled T2DM, which may have resulted in a lower baseline HbA1c and subsequent impact on the external validity of the results. Fourth, this study used an absolute exercise intensity for the intervention, therefore some participants could have exercised at a lower or higher relative intensity of exercise [49]. However, each participant was reassessed every four weeks to adjust their exercise intensity to account for improvements in fitness. Although several limitations exist, this study is strengthened by the use of strong measurement tools for cardiorespiratory fitness and body composition, as well as the gold standard measurement for glycemia–performed twice at each time point to account for additional measurement error. Further, the exercise intervention also reflected the Canadian Physical Activity Guidelines of 150 minutes of moderate-to-vigorous intensity aerobic physical activity a week [50]. In addition, an exercise professional fully supervised all sessions for 28 weeks to ensure participants were exercising at the appropriate intensity. Finally, the statistical analyses were adjusted for T2DM medications in the HbA1c analysis.

In conclusion, short- and long-duration T2DM did not differently impact the efficacy of a 28-week moderate-to-vigorous intensity aerobic exercise intervention. Furthermore, no significant interactions of time and T2DM duration existed for any body composition measures, cardiorespiratory fitness, or glycemia. However, our findings support that a supervised, aerobic exercise intervention can improve body composition measures and cardiorespiratory fitness in individuals with short- and long-duration T2DM, and that some of these changes can be associated with improved glycemia for those with long-duration T2DM.
